# Unrecognized Non-Q-Wave Myocardial Infarction: Prevalence and Prognostic Significance in Patients with Suspected Coronary Disease

**DOI:** 10.1371/journal.pmed.1000057

**Published:** 2009-04-21

**Authors:** Han W. Kim, Igor Klem, Dipan J. Shah, Edwin Wu, Sheridan N. Meyers, Michele A. Parker, Anna Lisa Crowley, Robert O. Bonow, Robert M. Judd, Raymond J. Kim

**Affiliations:** 1 Duke Cardiovascular Magnetic Resonance Center, Division of Cardiology, Duke University, Durham, North Carolina, United States of America; 2 Feinberg Cardiovascular Research Institute, Division of Cardiology, Northwestern University, Chicago, Illinois, United States of America; University of Sydney, Australia

## Abstract

**Background:**

Unrecognized myocardial infarction (UMI) is known to constitute a substantial portion of potentially lethal coronary heart disease. However, the diagnosis of UMI is based on the appearance of incidental Q-waves on 12-lead electrocardiography. Thus, the syndrome of non-Q-wave UMI has not been investigated. Delayed-enhancement cardiovascular magnetic resonance (DE-CMR) can identify MI, even when small, subendocardial, or without associated Q-waves. The aim of this study was to investigate the prevalence and prognosis associated with non-Q-wave UMI identified by DE-CMR.

**Methods and Findings:**

We conducted a prospective study of 185 patients with suspected coronary disease and without history of clinical myocardial infarction who were scheduled for invasive coronary angiography. Q-wave UMI was determined by electrocardiography (Minnesota Code). Non-Q-wave UMI was identified by DE-CMR in the absence of electrocardiographic Q-waves. Patients were followed to determine the prognostic significance of non-Q-wave UMI. The primary endpoint was all-cause mortality. The prevalence of non-Q-wave UMI was 27% (50/185), compared with 8% (15/185) for Q-wave UMI. Patients with non-Q-wave UMI were older, were more likely to have diabetes, and had higher Framingham risk than those without MI, but were similar to those with Q-wave UMI. Infarct size in non-Q-wave UMI was modest (8%±7% of left ventricular mass), and left ventricular ejection fraction (LVEF) by cine-CMR was usually preserved (52%±18%). The prevalence of non-Q-wave UMI increased with the extent and severity of coronary disease on angiography (*p*<0.0001 for both). Over 2.2 y (interquartile range 1.8–2.7), 16 deaths occurred: 13 in non-Q-wave UMI patients (26%), one in Q-wave UMI (7%), and two in patients without MI (2%). Multivariable analysis including New York Heart Association class and LVEF demonstrated that non-Q-wave UMI was an independent predictor of all-cause mortality (hazard ratio [HR] 11.4, 95% confidence interval [CI] 2.5–51.1) and cardiac mortality (HR 17.4, 95% CI 2.2–137.4).

**Conclusions:**

In patients with suspected coronary disease, the prevalence of non-Q-wave UMI is more than 3-fold higher than Q-wave UMI. The presence of non-Q-wave UMI predicts subsequent mortality, and is incremental to LVEF.

**Trial Registration:**

Clinicaltrials.gov NCT00493168

## Abstract 2

Using delayed-enhancement cardiovascular magnetic resonance, Han Kim and colleagues show that in patients with suspected coronary disease the prevalence of unrecognized myocardial infarction without Q-waves is more than 3-fold higher than that with Q-waves and predicts subsequent mortality.

## Editors' Summary

**Background**

Coronary artery disease (CAD; also called coronary heart disease) is the leading cause of death among adults in developed countries. In the USA alone, it kills nearly half a million people every year. CAD is caused by narrowing of the coronary arteries, the blood vessels that supply the heart with oxygen and nutrients. With age, fatty deposits (atherosclerotic plaques) coat the walls of these arteries and restrict the heart's blood supply, which causes the characteristic symptoms of CAD—angina (chest pains that are usually relieved by rest) and shortness of breath. In addition, if a plaque breaks off the wall of a coronary artery, it can completely block that artery and kill part of the heart, which causes a potentially fatal heart attack (doctors call this a myocardial infarction or MI). Heart attacks are often characterized by long-lasting chest pain that is not relieved by rest. Risk factors for CAD include smoking, high blood pressure, high blood levels of cholesterol (a type of fat), and being overweight. Treatments for the condition include lifestyle changes (for example, losing weight), and medications that lower blood pressure and blood cholesterol. The narrowed arteries can also be widened using a device called a stent or surgically bypassed.

**Why Was This Study Done?**

Not everyone who has a heart attack has chest pain. In fact, some studies suggest that 40–60% of MIs have no obvious symptoms. It is important, however, that these “unrecognized” MIs (UMIs) are diagnosed because they have death rates similar to those of MIs with clinical symptoms and need to be treated in a similar way. Traditionally, UMIs have been diagnosed using an electrocardiogram (ECG). When the heart beats, it generates small electric waves that can be picked up by electrodes attached to the skin. The pattern of these waves (the ECG) provides information about the heart's health. Alterations in the ECG, leading to so-called Q-waves, indicate that a UMI has occurred some time previously. However, not all UMIs result in Q-waves. In this study, the researchers use a recently developed technique—delayed enhancement cardiovascular magnetic resonance (DE-CMR), which can detect heart damage even in patients whose Q-waves are absent—to measure the prevalence (the fraction of a population that has a disorder) of non-Q-wave UMI. The researchers also investigate whether non-Q-wave UMI increases the risk of death.

**What Did the Researchers Do and Find?**

The researchers used electrocardiography and DE-CMR to look for Q-wave and non-Q-wave UMI, respectively, in 185 patients with suspected CAD but no history of MI. They then followed the patients for 2 years to discover whether a diagnosis of non-Q-wave UMI predicted their likelihood of dying from any cause or from a heart problem. 27% of the patients had evidence of non-Q-wave UMI whereas only 8% had evidence of Q-wave UMI. Patients with non-Q-wave UMI tended to have only a small area of heart damage and, consistent with this limited damage, their hearts pumped near-normal volumes of blood. Examination of the patients' arteries with a technique called coronary angiography indicated that the patients with widespread and/or severe CAD had a higher prevalence of non-Q-wave UMI than those with limited CAD. Finally, patients with non-Q-wave UMI had an 11-fold higher risk of death from any cause and a 17-fold higher risk of death from a heart problem than patients without UMI.

**What Do These Findings Mean?**

These findings indicate that non-Q-wave UMI occurs more than 3-times as often in patients with suspected CAD than Q-wave UMI and that patients with non-Q-wave UMI have a much greater risk of dying than patients without MI. Thus, if all cases of UMI—both Q-wave and non-Q-wave UMI—could be identified, it might be possible to reduce the number of deaths among people with CAD. However, before any recommendations are made to include DE-CMR in the routine examination of people with suspected CAD to achieve this aim, additional studies must be undertaken to confirm that non-Q-wave UMI is a common feature of CAD and to test whether the early diagnosis of non-Q-wave UMI does extend the life expectancy of people with CAD.

**Additional Information**

Please access these Web sites via the online version of this summary at http://dx.doi.org/10.1371/journal.pmed.1000057.

- This study is further discussed in a *PLoS Medicine* Perspective by Clara Chow
- The MedlinePlus encyclopedia has pages on coronary heart disease, heart attacks, and electrocardiograms (in English and Spanish). MedlinePlus also provides links to further information on all aspects of heart disease (in English and Spanish)
- Information is available from the US National Heart Lung and Blood Institute on coronary heart disease
- The UK National Health Service Choices website also provides information about coronary heart disease (in several languages).
- The Nobel Foundation provides an interactive electrocardiogram game

## Introduction

In patients with coronary artery disease (CAD), the diagnosis of myocardial infarction (MI) directs clinical management and affects prognosis. However, MI can be associated with atypical, minimal, or no symptoms and thus may occur “unrecognized” by the patients themselves and their physicians. Despite this unremarkable presentation, unrecognized MI (UMI) adversely affects prognosis with mortality rates that are similar to myocardial infarctions that are recognized clinically [1]–[3].

Large population surveys have shown that as many as 40%–60% of all MIs are unrecognized [1]–[6]. In these studies, serial 12-lead electrocardiograms (ECGs) were performed annually or biennially and the diagnosis of UMI was made on the basis of new Q-waves on successive ECGs without the occurrence of a clinically evident MI. Therefore, by definition, patients with non-Q-wave infarcts were not identified. Accordingly, the syndrome of non-Q-wave UMI has not been examined, and the prevalence and prognostic significance of this syndrome are unknown.

Delayed-enhancement cardiovascular magnetic resonance (DE-CMR) is a relatively recent technique that can identify MI, even when small, subendocardial, or without associated Q-waves [7]. Initial studies suggest that DE-CMR for the assessment of MI is reproducible [8] and provides results superior to radionuclide imaging in patients with small infarcts [9], [10].

The purpose of the current study was to determine the prevalence of non-Q-wave UMI—identified by DE-CMR—in comparison to Q-wave UMI in patients with suspected CAD who had no history of MI. We prospectively enrolled patients scheduled for invasive coronary angiography in order to directly relate the presence of UMI to the extent and severity of coronary atherosclerosis. Patients were then followed for over 2 y to determine whether the presence of non-Q-wave UMI portends increased mortality.

## Methods

### Population

Patients with suspected CAD (i.e., not already known to have CAD) who were scheduled for elective X-ray coronary angiography were prospectively recruited. The decision to perform coronary angiography had been made prior to study recruitment. Prior MI was defined using the criteria of the World Health Organization (WHO criteria: [1] evolving diagnostic ECG, or [2] diagnostic ECG and abnormal enzymes, or [3] prolonged cardiac pain and abnormal enzymes). Patients with MI verified by the medical record were excluded. Patients with only Q-waves on 12-lead ECG in the absence of clinical MI were not excluded, since one of the main aims of the study was to compare the prevalence of non-Q-wave to that of Q-wave UMI.

Additional exclusion criteria were: (1) history of percutaneous coronary intervention (PCI) or coronary artery bypass grafting (CABG), since small infarcts are common in the setting of successful revascularization procedures [11] and we wished to exclude infarcts from iatrogenic causes; (2) nonischemic myocardial disorders such as hypertrophic or infiltrative cardiomyopathy, and myocarditis, since these disorders frequently cause myocardial necrosis and/or scarring [12]–[14]; (3) any serious intercurrent illness such as uncured malignancy that could shorten survival to less than 2 y; or (4) contraindication to CMR (e.g., pacemaker). All patients were enrolled before the recent Federal Drug Administration alerts regarding the rare occurrence of nephrogenic systemic fibrosis associated with gadolinium administration [15]. Four patients had end-stage renal disease and were receiving dialysis therapy at the time of enrollment. None of the study participants developed nephrogenic systemic fibrosis during the follow-up period.

Patients were prospectively enrolled from two sites, Northwestern Memorial Hospital (*n* = 100) between January 1998 through October 2001 and Duke University Medical Center (*n* = 85) between December 2002 and January 2004. The gap in study enrollment occurred during relocation of some personnel to the latter institution. Institutional review board approval was obtained at both sites. Potential participants were identified prospectively from the cardiac catheterization laboratory schedule and subsequently contacted by the investigators. Those who fulfilled the study criteria were asked to participate, and consecutive patients who signed consent were included in the study. Importantly, all CMR studies were performed only for research purposes and were not clinically ordered scans. There were no significant incidental findings, and CMR results were not used to guide clinical decision-making (e.g., coronary revascularization).

### Protocol

#### Baseline procedures

All patients were interviewed using a standardized questionnaire to obtain a complete medical history, including responses to the Rose chest pain questionnaire [16]. Participants were considered to have hypertension, diabetes mellitus, or hypercholesterolemia if they had a documented diagnosis by a physician, supportive laboratory data, or were taking medications for these conditions. Coronary heart disease risk was estimated by the Framingham prediction algorithm [17]. Standard 12-lead ECGs were obtained in all patients. In the majority of patients, CMR was performed immediately prior to angiography on the same day (interquartile range [IQR] 0–2 d; within 30 d in all patients).

#### Follow-up

Clinical follow-up was obtained annually via telephone interview with the patient or immediate family member, the patient's physician, hospital records, and death certificates. Data regarding subsequent revascularization, MI, and death were recorded. The prespecified primary endpoint was all-cause mortality [18]. The secondary endpoint was cardiac mortality. The median follow-up time at Northwestern was similar to that at Duke (2.2 versus 2.4 y, respectively, *p* = 0.22). All participants (survivors) had a minimum of 1 y of follow-up.

### CMR

All images were acquired on a clinical 1.5T scanner (Siemens Sonata) using a phased array receiver coil during repeated breath-holds as described previously [19]. Briefly, cine-CMR images were acquired in multiple short- and long-axis views using a steady-state free procession sequence. Short-axis views were obtained every 1 cm to cover the entire left ventricle (6 mm thickness, 4 mm gap). A gadolinium-based contrast agent (gadoteridol or gadoversetamide) was administered intravenously (0.15 mmol/kg), and DE-CMR images were acquired in the same views used for cine-CMR 10–15 min later. DE-CMR images were acquired using a segmented inversion-recovery sequence with inversion time adjusted to null normal myocardium; typical in-plane resolution was 1.9×1.4 mm with a slice thickness of 6.0 mm [20]. No patient was excluded on the basis of CMR image quality.

### Analysis

Cine-CMR, DE-CMR, ECG, and coronary angiography were interpreted by a consensus of two observers who were masked to patient identity and clinical history in separate reading sessions.

#### Cine CMR

Left ventricular ejection fraction (LVEF) and volumes were quantitatively measured via end-diastolic and end-systolic endocardial contours from the stack of short-axis cine images [19].

#### DE-CMR

The presence and location of hyperenhanced tissue, which was assumed to represent scarred myocardium [19], [21], was determined by visual inspection using the AHA 17-segment model [22]. Regional enhancement was scored according to the spatial extent of hyperenhanced tissue within each segment (0 = no hyperenhancement; 1 = 1%–25% hyperenhanced; 2 = 26%–50%; 3 = 51%–75%; and 4 = 76%–100%) [7]. On a per patient basis, infarction was considered transmural if one or more segments had a score of 4 (76%–100%). Additionally, the pattern of hyperenhancement was classified as either CAD-type or non-CAD-type as described previously [23]–[25]. In brief, since ischemic injury progresses as a “wavefront” from the subendocardium to the epicardium [26], hyperenhancement involving the subendocardium was considered CAD-type. Conversely, hyperenhancement patterns that spared the subendocardium and instead were limited to the middle or epicardial portion of the left ventricular (LV) wall were considered non-CAD-type. An exception was in the setting of subendocardial enhancement diffusely throughout the entire LV, which can occur in certain nonischemic cardiomyopathies (e.g., cardiac amyloidosis). This pattern was classified as non-CAD-type.

Although our primary focus was to identify the presence of UMI, infarct size was also measured by planimetry from the stack of short-axis DE-CMR images [27]. The infarct borders were determined visually in our CMR core laboratory. Interobserver agreement for infarct size, routinely tested as part of our core laboratory services using Bland-Altman analysis, demonstrated a bias of 1.0% with a standard deviation (SD) of the difference of 2.6%. Additionally, in a random sample of 15% of the study population, infarct size was measured twice (6 mo apart) to determine intraobserver agreement. Analysis of this subset demonstrated a bias of −0.1% with a standard deviation of the difference of 0.8%. A fixed cutoff of 2 SD above the mean signal intensity of normal myocardium was not used to define the infarcted region because this approach does not account for partial volume effects [27].

#### ECG

The presence of Q-waves was determined on the basis of Minnesota codes 1-1-1 to 1-2-7 [28]. Electrocardiograms were also scored for the presence of complete left bundle branch block (7-1-1).

#### Coronary angiography

Obstructive CAD was defined as ≥50% narrowing of the luminal diameter of at least one major epicardial artery [29]. Luminal narrowing was estimated visually by the consensus of two experienced readers.

#### Definitions of Q-wave and non-Q-wave UMI

Q-wave UMI was defined solely by the presence of major Q-waves on electrocardiography to allow a direct comparison of the results of the current study to published data [2]–[4]. Non-Q-wave UMI was defined by the presence of CAD-type hyperenhancement on DE-CMR in those patients lacking Q-waves. Patients with non-CAD-type hyperenhancement and those without hyperenhancement were both classified as having “no MI.” Since these two groups may have different prognoses, survival in the “no MI” group was also assessed after excluding patients with non-CAD-type hyperenhancement.

### Statistical Analysis

Continuous data are presented as mean±SD or, in cases where the distribution is not normal, as median and IQR. Two-sample *t*-tests were used to compare mean values of continuous data between two groups. Comparisons between discrete data were made using Chi-square tests. Differences in means between more than two groups were assessed using analysis of variance; the Bonferroni method of adjustment was used in making multiple pairwise comparisons. The relationship between angiographic parameters and the frequency of Q-wave and non-Q-wave UMI was evaluated using the Chi-square test for trend.

In order to identify the clinical characteristics associated with non-Q-wave UMI, univariable and multivariable logistic regression analyses were performed in the remaining cohort of patients after excluding those with Q-wave UMI. In the same group, Cox regression analysis was performed to assess the effect of non-Q-wave UMI on both all-cause and cardiac mortality. Initially, the univariable predictors were identified, then only variables with *p*-values below 0.10 were considered candidate predictors of mortality in the multivariable model to reduce the possibility of overfitting. As part of separate analyses, revascularization (CABG or PCI) during the follow-up period and enrollment site were included as covariates in order to account for their effects on survival. All statistical tests were two-tailed and *p*<0.05 was regarded as significant.

## Results

### Clinical Characteristics and Prevalence of Unrecognized Non-Q-wave MI

A total of 185 patients were enrolled. All successfully underwent CMR. Table 1 demonstrates the clinical characteristics of the population. Overall, the mean age of the group was 60.4 y (range 25–86 y). Left ventricular function was usually preserved (LVEF 59%±18%). The majority had chest pain (62%) and/or dyspnea (30%). In 158 patients (85%), the indication for coronary angiography was a positive or equivocal radionuclide, echocardiography, or treadmill stress test. In the remaining 15%, the primary physician elected to proceed directly to coronary angiography based on typical symptoms alone.

**Table 1:** Clinical characteristics.

Characteristic	All Patients (*n* = 185)	Q-wave UMI (*n* = 15)	Non-Q-wave UMI (*n* = 50)	No MI (*n* = 120)	*p*-Value
**Age**	60.4±11.2	58.5±10.7	64.3±11.4	59.1±11.0	**0.02** a
**Male**	66%	73%	74%	63%	0.30
**CAD risk factors**					
Hypertension	123 (66%)	12(80%)	35 (70%)	76 (63%)	0.36
Hypercholesterolemia	90 (49%)	7 (47%)	23 (46%)	60 (50%)	0.88
Cigarette smoking	53 (29%)	8 (53%)	16 (32%)	29 (24%)	0.052
Diabetes mellitus	57 (31%)	5 (33%)	22 (44%)	30 (25%)	**0.049** a
Family history of CAD	66 (36%)	2 (13%)	16 (30%)	49 (41%)	0.07
Number of risk factors	2.1±1.1	2.3±1.3	2.2±1.1	2.0±1.1	0.49
**Symptoms**					
Chest pain^b^					0.74
Typical angina	57 (31%)	4 (27%)	16 (32%)	37 (31%)	
Atypical angina	57 (31%)	6 (43%)	12 (24%)	39 (33%)	
None	71 (38%)	5 (36%)	22 (43%)	44 (37%)	
Dyspnea	56 (30%)	4 (27%)	19 (38%)	33 (28%)	0.38
NYHA class	1.4±0.8	1.5±1.0	1.7±1.0	1.3±0.7	0.051
**Medications**					
Aspirin	109 (59%)	13 (87%)	32(64%)	64 (53%)	**0.03**
Beta blocker	74 (40%)	7 (47%)	21 (42%)	46 (38%)	0.78
ACE-I	86 (46%)	7 (47%)	27 (54%)	52 (43%)	0.45
Statin	65 (35%)	2 (13%)	19 (38%)	44 (37%)	0.18
Nitrate	29 (16%)	6 (40%)	13 (26%)	10 (8%)	**0.0004** a
Calcium channel blocker	38 (21%)	5 (33%)	16 (32%)	17 (14%)	**0.01** a
**Framingham risk score** c	16.2±12.3	20.1±13.6	22.5±15.3	13.5±10.0	**0.0006** a
**12-lead ECG**					
Q-waves^d^	15 (8%)	15 (100%)	—	—	—
Left bundle branch block^e^	10 (5%)	—	4 (8%)	6 (5%)	—
**Cine CMR**					
LVEF	59±18	48±20	52±18	63±17	**<0.0001** a
End diastolic volume, ml	114±47	104±36	106±45	118±48	0.23
End systolic volume, ml	50±41	56±35	54±37	48±43	0.57

a *p*<0.05 for pairwise comparison between non-Q-wave UMI and no MI.

b Angina defined by Rose Chest Pain Questionnaire. The p value pertains to the comparison in the distribution of patients according to chest pain.

c Calculated in the 140 patients who had all relevant blood tests.

d Minnesota codes 1-1-1 to 1-2-7.

e Minnesota codes 7-1-1.

ACE-I, angiotensin converting enzyme inhibitor.

Overall, the prevalence of non-Q-wave UMI was 27% (50/185) and 3.3-fold higher than that of Q-wave UMI (8%, 15/185). Patients with non-Q-wave UMI were older, had a higher prevalence of diabetes, had a higher Framingham risk, and had lower LVEFs than those without MI (Table 1).

### Infarct Size and Location

In patients with non-Q-wave UMI, infarct size varied over a wide range, but overall was relatively modest (8%±7% of LV; range 1%–22%). Patients with Q-wave UMI also had a wide range of infarct sizes (0%–27%), including three without evidence of infarction by DE-CMR. All three had normal coronary angiograms, suggesting that the Q-waves in these three were false positive for MI. Mean infarct size excluding these three patients was 14%±9% of LV, and larger than for non-Q-wave UMI patients (*p* = 0.02). Additionally, patients with non-Q-wave UMI were less likely to have transmural infarcts than those with Q-wave UMI (18% versus 58%, respectively, *p* = 0.002). In patients with non-Q-wave UMI, infarct location by DE-CMR was distributed as follows: 40% left anterior descending coronary artery (LAD) perfusion territory, 47% right coronary artery (RCA), and 13% left circumflex coronary artery (LCx). The distribution was similar (*p* = 0.74) in those with Q-wave UMI (44% LAD, 50% RCA, 6% LCx).

**Figure 1:**
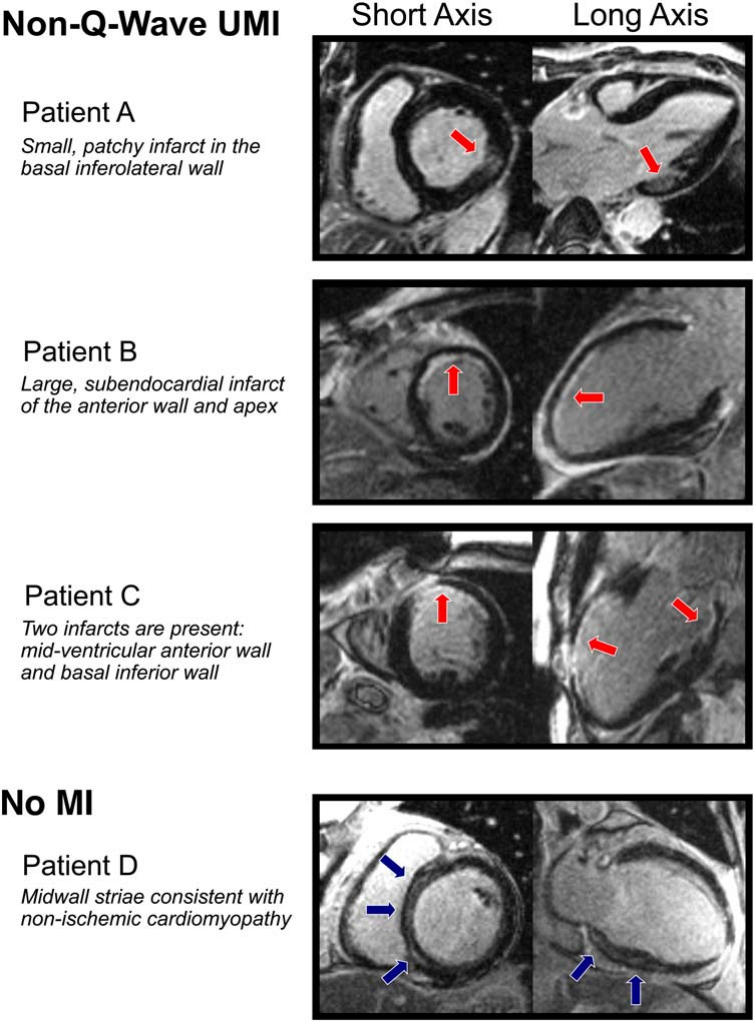
Typical DE-CMR images. Short and long axis views of DE-CMR images from four patients are shown. Patients A–C demonstrate hyperenhancement (red arrows) consistent with prior myocardial infarction. None had Q-waves on electrocardiography, and all three were classified as having non-Q-wave UMI. Of note, Patient C has evidence of two distinct infarcts. Patient D has hyperenhancement (blue arrows) involving the midwall of the interventricular septum, sparing the subendocardium. This pattern is not consistent with prior myocardial infarction, and this patient was categorized in the “no MI” group. See text for further details.

Ten patients (20%) with non-Q-wave and four (28%) with Q-wave UMI had two infarcts (i.e., separate infarcts in different coronary artery territories). Typical images of patients with non-Q-wave UMI are shown in Figure 1 (patients A–C). In nine patients (5% of the overall population), hyperenhancement in a non-CAD-type pattern was observed, the most common of which was midwall striae in the interventricular septum (*n* = 6, Figure 1, patient D).

### Correlation with Coronary Artery Disease

**Figure 2:**
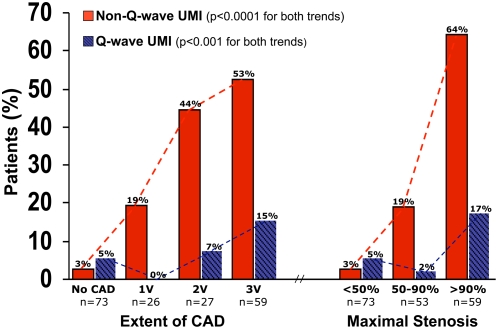
Prevalence of UMI stratified by angiographic extent and severity of coronary artery disease. The prevalence of non-Q-wave and Q-wave UMI increased with both the extent and severity of CAD. See text for further details. CAD = coronary artery disease; UMI = unrecognized myocardial infarction; 1V = single vessel; 2V = double vessel; 3V = triple vessel.

Overall, coronary angiography revealed obstructive CAD in 61% (112/185) of the population. In patients with non-Q-wave UMI, with Q-wave UMI, and without MI, CAD was present in 96% (48/50), 73% (11/15), and 44% (53/120), respectively. Figure 2 shows the prevalence of UMI stratified by the angiographic extent and severity of CAD. Both the prevalence of non-Q-wave and Q-wave UMI increased with CAD extent (*p*<0.0001 and *p*<0.001 for trend, respectively) and severity (*p*<0.0001 and *p*<0.001 for trend, respectively). However, the relationship was steeper for non-Q-wave UMI, with the prevalence reaching 53% in patients with triple vessel disease (versus 15% Q-wave UMI prevalence) and 64% in those with maximal stenosis over 90% (versus 17% Q-wave UMI prevalence).

### Clinical Predictors of Non-Q-Wave UMI

Besides the correlation with angiographic CAD extent and severity, the following were significant univariable predictors of non-Q-wave UMI: age, diabetes, NHYA class, Framingham risk, and LVEF (Table 2). Multivariable analysis demonstrated that age (*p* = 0.01), diabetes (*p* = 0.03), and LVEF (*p* = 0.0003) remained independent clinical predictors. The risk of non-Q-wave UMI increased by 1.6-fold, 2.4-fold, and 1.4-fold for every 10-y increase in age, for the presence of diabetes, and for every 10-percentage point decrease in LVEF, respectively.

**Table 2:** Clinical predictors of non-Q-wave UMI.

Characteristic	Univariable		Multivariable	
	Odds Ratio^a^ (95% CI)	*p*-Value	Odds Ratio^a^ (95% CI)	*p*-Value
**Age**	**1.05 (1.01–1.08)**	**0.007**	**1.05 (1.01–1.08)**	**0.01**
**Male**	1.71 (0.82–3.55)	0.15		
**CAD risk factors**				
Hypertension	1.35 (0.66–2.75)	0.41		
Hypercholesterolemia	0.85 (0.44–1.65)	0.85		
Cigarette smoking	1.48 (0.71–3.05)	0.30		
Diabetes mellitus	**2.36 (1.18–4.72)**	**0.02**	**2.40 (1.12–5.11)**	**0.03**
Family history of CAD	0.62 (0.31–1.26)	0.19		
Number of risk factors	1.18 (0.86–1.62)	0.30		
**Symptoms**				
Chest pain^b^				
Typical angina	1.06 (0.52–2.15)	0.88		
Atypical angina	0.66 (0.31–1.39)	0.27		
Any	0.74 (0.38–1.44)	0.37		
Dyspnea	1.62 (0.80–3.25)	0.18		
NYHA class	**1.64 (1.09–2.45)**	**0.02**		
**Framingham risk score** c	**1.06 (1.02–1.09)**	**0.0009**		
**12-lead ECG**				
Left bundle branch block^d^	1.65 (0.45–6.13)	0.45		
**Cine CMR**				
LVEF	**0.97 (0.95–0.99)**	**0.0008**	**0.96 (0.94–0.98)**	**0.0003**
End diastolic volume	0.99 (0.99–1.00)	0.15		
End systolic volume	1.00 (1.00–1.01)	0.39		

a The odds ratios are associated with a single unit increase for all of the continuous variables.

b Angina defined by Rose Chest Pain Questionnaire.

c Calculated in the 130 patients who had all relevant blood tests.

d Minnesota codes 7-1-1.

### Survival

The median follow-up time was 2.2 y (IQR 1.8–2.7). No patient was lost to follow-up. During the follow-up period, 16 patients died, resulting in an overall mortality rate of 3.8% per year. Three patients experienced nonfatal MI during the follow-up period, and subsequently died 3, 4, and 28 mo later. Among patients with non-Q-wave UMI, there were 13 deaths (10.8% per year), including 10 cardiac, one noncardiac, and two from unknown causes. In patients with Q-wave UMI, there was one death (2.7% per year), which was cardiac. In patients without MI, there were two deaths (0.8%per year), including one cardiac and one noncardiac. Among the nine with non-CAD-type hyperenhancement, no deaths occurred.

**Figure 3:**
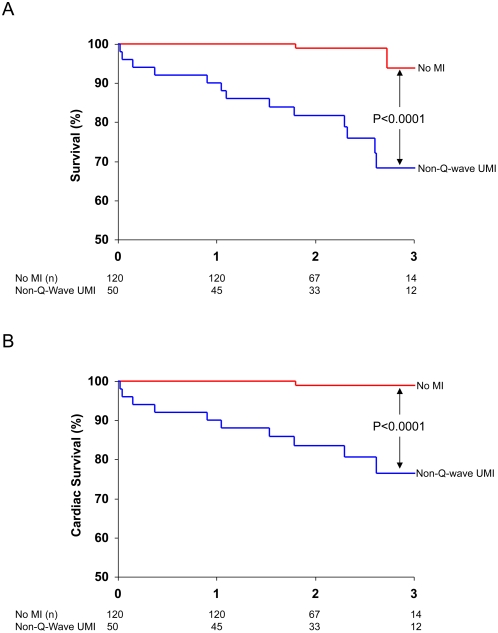
Kaplan–Meier estimates of survival (A) and cardiac survival (B) in patients with unrecognized non-Q-wave MI (blue line) and without MI (red line). Overall and cardiac survival in patients with non-Q-wave UMI was significantly reduced in comparison to patients without MI (*p*<0.0001 for both). The annual mortality in patients with non-Q-wave MI was 15-fold higher than that in patients without MI (10.8% per year versus 0.8% per year, respectively).

Patients with non-Q-wave UMI (*n* = 50) had reduced overall and cardiac survival compared to patients without MI (*n* = 120), as demonstrated by the Kaplan-Meier curves in Figure 3 (*p*<0.0001 for both). Given that the Q-wave UMI group consisted of only 15 patients, survival in this group was not compared with either the non-Q-wave UMI or the no-MI group. Among the clinical characteristics, significant univariable predictors of all-cause mortality were New York Heart Association (NYHA) class, LVEF, and non-Q-wave UMI (Table 3). However, in multivariable analysis—in which only candidate variables with *p*<0.10 from the univariable analysis were considered (NYHA class, LVEF, non-Q-wave UMI, and revascularization during the follow-up period)—LVEF and non-Q-wave UMI were independent predictors. We note that 35% (64/185) of the entire study population underwent revascularization during the follow-up period (CABG, 46; PCI, 18), and specifically in the two comparison groups, 66% (33/50) of patients with non-Q-wave UMI and 20% (24/120) without MI underwent revascularization. After adjustment for revascularization, multivariable analysis demonstrated that LVEF and the presence of non-Q-wave UMI remained as independent predictors of mortality. These two variables were again identified as the only independent predictors after adjustment for enrollment site. Likewise, when only fatal cardiac events were considered, LVEF and non-Q-wave UMI were once again identified as independent predictors of mortality (Table 4).

**Table 3:** Predictors of all-cause mortality.

Variables	Univariable		Multivariable	
	Hazard Ratio (95% CI)	*p*-Value	Hazard Ratio (95% CI)	*p*-Value
**Baseline characteristics**				
Age	1.02 (0.98–1.07)	0.32		
Male	0.7 (0.2–1.8)	0.41		
Hypertension	3.2 (0.7–14.1)	0.13		
Hypercholesterolemia	0.9 (0.3–2.5)	0.85		
Cigarette smoking	1.8 (0.6–4.9)	0.29		
Diabetes mellitus	1.9 (0.7–5.2)	0.23		
Family history of CAD	0.6 (0.2–2.0)	0.41		
Number of risk factors	1.4 (0.8–2.2)	0.22		
Typical angina	0.99 (0.3–2.9)	0.98		
Atypical angina	0.4 (0.1–1.7)	0.38		
Dyspnea	1.9 (0.7–5.2)	0.22		
NYHA class	**1.7 (1.02–2.7)**	**0.04**		
Framingham risk	1.02 (0.98–1.05)	0.43		
**CINE CMR**				
LVEF	**0.97 (0.94–0.99)**	**0.004**	**0.97 (0.95–0.99)** a	**0.04** a
End diastolic volume	1.0 (0.98–1.01)	0.56		
End systolic volume	1.0 (0.99–1.01)	0.50		
**DE-CMR**				
Non-Q-wave UMI	**14.1 (3.2–62.7)**	**0.0005**	**11.4 (2.5–51.1)** a	**0.002** a
**Revascularization during the follow-up period**	2.5 (0.9–7.1)	0.09		

a After adjustment for revascularization during the follow-up period, the hazard ratio for the presence of non-Q-wave UMI was 9.9 (95% CI 2.0–48.2), *p* = 0.005. The hazard ratio of LVEF was 0.97 (95% CI 0.94–0.99), *p* = 0.04.

**Table 4:** Predictors of cardiac mortality.

Variables	Univariable		Multivariable	
	Hazard Ratio (95% CI)	*p*-Value	Hazard Ratio (95% CI)	*p*-Value
**Baseline characteristics**				
Age	1.01 (0.96–1.07)	0.68		
Male	0.6 (0.2–1.9)	0.34		
Hypertension	2.3 (0.5–10.6)	0.29		
Hypercholesterolemia	1.3 (0.4–4.1)	0.72		
Cigarette smoking	1.03 (0.3–3.9)	0.97		
Diabetes mellitus	1.8 (0.6–6.0)	0.32		
Family history of CAD	0.6 (0.2–2.3)	0.46		
Number of risk factors	1.3 (0.7–2.2)	0.44		
Typical angina	0.8 (0.2–2.9)	0.71		
Atypical angina	0.2 (0.03–1.8)	0.17		
Dyspnea	2.7 (0.8–8.9)	0.10		
NYHA class	**1.9 (1.1–3.2)**	**0.02**		
Framingham risk	0.99 (0.94–1.05)	0.82		
**CINE CMR**				
LVEF	**0.96 (0.93–0.99)**	**0.004**	**0.97 (0.94–0.99)** a	**0.04** a
End diastolic volume	1.0 (0.99–1.01)	0.96		
End systolic volume	1.0 (0.99–1.02)	0.28		
**DE-CMR**				
Non-Q-wave UMI	**23.1 (3.0–180.6)**	**0.003**	**17.4 (2.2–137.4)** a	**0.007** a
**Revascularization during the follow-up period**	3.2 (0.93–10.9)	0.07		

a After adjustment for revascularization during the follow-up period, the hazard ratio for the presence of non-Q-wave UMI was 14.3 (95% CI 1.7–124.4), *p* = 0.02. The hazard ratio of LVEF was 0.96 (95% CI 0.93–0.99), *p* = 0.03.

## Discussion

This investigation is, to our knowledge, the first to systematically characterize the syndrome of unrecognized non-Q-wave myocardial infarction and demonstrate its prognostic importance. In a cohort without known prior MI referred for coronary angiography, one-third (62/185) had evidence of unrecognized MI by DE-CMR, among whom 80% (50/62) had no Q-waves. The presence of unrecognized non-Q-wave MI predicted a 11-fold higher risk of death and a 17-fold higher risk of cardiac death than those without MI.

It has been estimated that 190,000 patients in the United States and perhaps as many as 300,000 in Europe suffer from silent MI annually [30], [31]. Since these estimates reflect only patients with electrocardiographic Q-waves, the data from the current study suggest that when those with non-Q-wave UMI are added, the actual incidence may be more than 3-fold higher. Although the magnitude of this discrepancy may be surprising, the concept that electrocardiography underestimates the incidence of UMI is not—since even among patients with clinically overt MI, Q-waves are more frequently absent than present [32]. Additionally, even if Q-waves are present initially, up to one-third may subsequently disappear during infarct healing [33].

The diagnosis of non-Q-wave UMI is difficult because, by definition, these patients either do not present during the acute phase of infarction or, even if they do present, MI is not suspected and cardiac biomarkers, such as troponins, are not drawn. Thus, even if some patients later undergo cardiac assessment, the infarct will be chronic. Accordingly, troponin levels will be normal, and the ECG will be nondiagnostic. In these circumstances, noninvasive imaging may be helpful.

Although radionuclide perfusion imaging and echocardiography have proven utility for the assessment of myocardial viability in patients with chronic CAD and extensive LV dysfunction [34], [35], viability testing is typically not performed in the setting of normal or mildly reduced LV function, and the presence of viability does not exclude subendocardial infarction [9], [10]. As far as we are aware, the sensitivity of these techniques for detecting chronic MI has not been tested. Moreover, there is a paucity of data from multicenter trials on the sensitivity of imaging approaches for the detection of MI, in either the acute or the chronic setting [36]. Recently, results from an international, multicenter trial evaluating DE-CMR for the detection of MI have been reported [36]. In this trial, the sensitivity of DE-CMR was tested in acute and chronic, and Q-wave and non-Q-wave MI patients. With appropriate contrast doses (0.2 mmol/kg or higher), DE-CMR was highly sensitive for the detection of acute and chronic Q-wave MI (99% and 98% respectively), and acute non-Q-wave MI (91%). The sensitivity was lowest in chronic non-Q-wave MI at 79%, likely reflecting the small mean infarct size in this cohort (6.8% of LV mass). Hence, while DE-CMR may be highly sensitive for detecting MI overall, identifying chronic non-Q-wave MI is challenging, which directly relates to the difficulty in diagnosing unrecognized non-Q-wave MI. The implication for the current study is that while 50 patients with non-Q-wave UMI were identified, this number may represent only 79% of the total number of patients with non-Q-wave UMI, and perhaps as many as 13 (21%) were not diagnosed.

One important prior study has examined the association of unrecognized myocardial scarring by DE-CMR and prognosis [37]. Kwong et al. reported that in a cohort of 195 patients, those with myocardial scarring had a more than 7-fold increased risk for major adverse cardiac events over those without [37]. This finding is consistent with the results of the current investigation, although Kwong et al. reported on all patients with UMI, and those with non-Q-wave UMI were not specifically investigated (e.g., clinical characteristics, predictors, angiographic features, etc.). Additionally, there appear to be notable differences in the two study populations. First, considerably more patients had UMI by DE-CMR than by ECG in the current study (62 versus 15, ratio 4.1) than in the study by Kwong et al. (44 versus 25, ratio 1.8), despite using the same Minnesota Code criteria for significant Q-waves. Second, when Q-waves were present, Kwong et al. reported that only 28% of patients had evidence of MI by DE-CMR, leading to a surprisingly high false positive rate of 72% for 12-lead electrocardiography. In contrast, in the current study, most patients with Q-waves (80%) demonstrated infarction by DE-CMR, and the false positive rate for electrocardiography was far smaller (20%). Third, cardiac mortality was quite high in the study by Kwong et al., at 6.6% per year overall and 22% per year in UMI patients (estimated from the reported hazard ratio). In comparison, the cardiac mortality that we observed was 2.9% per year overall and 6.9% per year in UMI patients. Hence, in the population studied by Kwong et al., cardiac mortality in those with UMI was over 3-fold higher. One potential explanation for these disparities may relate to how the participants were initially identified. Specifically, in the study by Kwong et al., all patients were referred for a clinically ordered CMR examination—a comprehensive and relatively uncommon test in comparison with echocardiography and radionuclide imaging. The cohort in which CMR is ordered may not be representative of those undergoing a standard noninvasive evaluation, and may include more patients with unusual clinical presentations and/or multiple cardiac issues. Additionally, in the study by Kwong et al., subsequent management decisions (e.g., whether or not to perform diagnostic cardiac catheterization or coronary revascularization) were in part dependent on the CMR results. In contradistinction, in the current study, participants were prospectively enrolled to undergo CMR only for the purpose of research, and clinical decisions were made without knowledge of the CMR findings.

The presence of non-Q-wave UMI was the strongest independent predictor of mortality in the current study. Patients with non-Q-wave UMI were older, more often diabetic, and had a higher Framingham risk than patients without MI. Not surprisingly, coronary atherosclerosis was extensive; multivessel disease occurred in 86% of non-Q-wave UMI patients. Hence, the poor prognosis associated with non-Q-wave UMI may relate to an abundance of clinical features that are associated with adverse outcomes. These features are also typically found in patients with clinically overt (recognized) non-Q-wave MI [32], who have poor outcomes—similar to or worse than those with overt Q-wave MI when including late mortality [32]. Thus, despite having different clinical presentations, patients with unrecognized non-Q-wave MI appear to share many similarities with patients presenting with overt non-Q-wave MI, in terms of both advanced atherosclerosis and poor prognosis. However, since the infarct was not diagnosed clinically, the poor prognosis observed in patients with unrecognized non-Q-wave MI may also be attributable in part to the absence of appropriate therapy for secondary prevention.

### Limitations

Beyond the presence of MI, infarct size may provide additional prognostic information in that larger infarcts are more frequently associated with ventricular tachyarrhythmias and sudden cardiac death [38], [39]. In the current study, the fit of the multivariable Cox regression model was not improved by substitution of infarct size for infarct presence/absence. However, this finding may be related to the small number of events in the study rather than a lack of incremental value for infarct size. Other studies have indicated that scarring in a non-CAD-type pattern, as occasionally found in nonischemic dilated cardiomyopathy, may also have prognostic importance [24]. In the current study, only nine patients had scarring in a non-CAD-type pattern, and none had events. One of the main aims of our investigation was to directly relate the presence of UMI with the extent and severity of coronary atherosclerosis. Accordingly, patients scheduled for X-ray coronary angiography were prospectively enrolled, and symptoms such as chest pain (62%) and dyspnea (30%) were fairly common. Thus, our findings may not be applicable to cohorts that are entirely asymptomatic. However, we note that in large population surveys such as the Framingham Heart Study, symptoms were present in nearly 50% of patients found to have Q-wave UMI [5], [6]. Additionally, we do not have data on the number of patients who were screened, declined to participate, or were excluded over the recruitment period. As a result, we are unsure if our cohort is fully representative of the population referred for angiography. However, the clinical profile of our study participants is similar to that of large population studies in which patients without prior history of MI underwent coronary angiography to exclude CAD [40], [41]. Thus, we believe that our cohort is representative of this specific population, which encompasses approximately one-third to one-half of all patients referred for invasive coronary angiography [40], [41].

### Summary

In patients with suspected coronary disease, the prevalence of non-Q-wave as compared with Q-wave UMI was more than 3-fold higher and was relatively common, occurring in over 25% of the study cohort. Patients with non-Q-wave UMI frequently had extensive coronary atherosclerosis and had more than a 11-fold higher risk of death than those without MI. Given the aging population and the increasing prevalence of diabetes, the ability to identify unrecognized MI may have important implications for individual patients and for public health policy recommendations, However, it remains untested if early diagnosis with appropriate MI treatment alters prognosis. Thus, clinical trials are needed to test this strategy, and then, depending on the findings, guidelines could be formulated for CMR referral.

## Abbreviations

CABG: coronary artery bypass grafting
CAD: coronary artery disease
CI: confidence interval
CMR: cardiovascular magnetic resonance
DE-CMR: delayed-enhancement cardiovascular magnetic resonance
ECG: electrocardiogram
HR: hazard ratio
LAD: left anterior descending coronary artery
LCx: left circumflex coronary artery
LV: left ventricle
LVEF: left ventricular ejection fraction
MI: myocardial infarction
NYHA: New York Heart Association
PCI: percutaneous coronary intervention
RCA: right coronary artery
UMI: unrecognized myocardial infarction
